# Perioperative Systemic Treatment Patterns and Postoperative Mortality Among Patients With Muscle‐Invasive Bladder Cancer Undergoing Radical Cystectomy in Japan: WAKKA‐MIBC Study

**DOI:** 10.1111/iju.70565

**Published:** 2026-07-25

**Authors:** Eiji Kikuchi, Jumpei Tokumaru, Daisuke Kawai, Ryotaro Ide, Hiroshi Komazaki, Hiroshi Kitagawa, Junichi Inokuchi

**Affiliations:** ^1^ Department of Urology St. Marianna University School of Medicine Kawasaki Kanagawa Japan; ^2^ Medical Department Astra Zeneca K.K. Tokyo Japan; ^3^ Department of Urology, Graduate School of Medicine University of the Ryukyus Okinawa Japan

**Keywords:** cystectomy, database, neoadjuvant therapy, retrospective studies, urinary bladder neoplasms

## Abstract

**Objectives:**

To characterize the temporal changes in perioperative systemic treatment patterns and postoperative mortality among Japanese patients with stage II–III muscle‐invasive bladder cancer (MIBC) undergoing radical cystectomy (RC) from 2013 to 2024.

**Methods:**

This retrospective observational cohort study assessed neoadjuvant chemotherapy (NAC) and adjuvant therapy (AT) relative to RC. Treatment patterns (NAC + RC + AT, NAC + RC, RC + AT, and RC alone), NAC regimen selection, and 30‐ and 90‐day postoperative mortality were evaluated across three periods (2013–2017, 2018–2021, and 2022–2024). Analyses were descriptive.

**Results:**

Among 5 208 patients, the use of NAC + RC increased from 31.4% in 2013–2017 to 50.4% in 2022–2024, and the use of NAC + RC + AT increased from 8.7% to 22.4%. RC alone decreased from 46.3% to 21.4%. Reduced‐dose gemcitabine + cisplatin (GC) and gemcitabine + carboplatin were frequently used and remained common throughout the study period. In 2022–2024, 18.8% of patients received adjuvant nivolumab; most had received NAC beforehand (242/270). The 30‐ and 90‐day mortality remained low regardless of the increase in NAC use (30‐day: ≤ 0.2% with NAC vs. ≤ 1.5% without; 90‐day: ≤ 1.4% with NAC vs. ≤ 3.3% without).

**Conclusions:**

Perioperative systemic therapy for MIBC in Japan has intensified over the past decade, with increased adoption of NAC and adjuvant nivolumab. Dose‐modified GC and carboplatin‐based regimens have remained commonly used throughout the study period. Even with increased NAC use, postoperative mortality has remained low.

**Abbreviations**
AT: adjuvant therapy
ATC: Anatomical Therapeutic Chemical
dd‐MVAC: dose‐dense methotrexate + vinblastine + doxorubicin + cisplatin
DPC: Diagnosis Procedure Combination
FAS: full analysis set
G‐carboplatin: gemcitabine + carboplatin
GC: gemcitabine + cisplatin
ICD‐10: International Classification of Diseases, 10th Revision
ICI: immune checkpoint inhibitor
LRC: laparoscopic radical cystectomy
MDV: Medical Data Vision
MIBC: muscle‐invasive bladder cancer
MVAC: methotrexate + vinblastine + doxorubicin + cisplatin
NAC: neoadjuvant chemotherapy
NMIBC: non‐muscle‐invasive bladder cancer
RARC: robot‐assisted radical cystectomy
RC: radical cystectomy
TNM: tumor–node–metastasis

## 1 Introduction

Bladder cancer is a common malignancy worldwide and ranks among the most frequently diagnosed cancers, particularly in men [1]. Incidence varies geographically, and significant increases have been observed in East Asia [2]. Approximately 25% of patients present with muscle‐invasive bladder cancer (MIBC) at initial diagnosis [3]. MIBC is associated with a substantially worse prognosis than non‐muscle‐invasive bladder cancer (NMIBC), with high risks of recurrence and disease‐specific mortality [4].

Outcomes for patients with MIBC treated with radical cystectomy (RC) alone remain insufficient, with 5‐year survival rates of approximately 50%–70% in historical series [4]. Neoadjuvant chemotherapies (NAC), such as gemcitabine + cisplatin (GC) and dose‐dense methotrexate + vinblastine + doxorubicin + cisplatin (dd‐MVAC), have proven effective and tolerable [5] and have become standard treatments in this setting.

In recent years, the use of NAC for MIBC has increased [6]. However, real‐world adoption of NAC in Japan has been limited [6, 7, 8], which may be related to concerns that NAC may delay definitive surgery, uncertain treatment response, the risk of disease progression during NAC, and the advanced age of patients, meaning they could frequently harbor comorbidities. Additionally, approximately 40% of patients with MIBC are ineligible for standard cisplatin‐based NAC due to impaired renal function and/or other comorbidities [9]. In 2022, the immune checkpoint inhibitor (ICI) nivolumab was approved as an adjuvant therapy (AT) for high‐risk patients with MIBC in Japan [10, 11]. The clinical landscape of RC has also evolved, including the adoption of robot‐assisted RC (RARC), which is associated with shorter hospital stays and lower rates of major postoperative complications compared with open RC [12], which may influence perioperative treatment selection.

However, the contemporary real‐world treatment landscape for NAC and AT in patients with MIBC in Japan remains unclear. Data regarding how perioperative regimens have changed following the introduction of nivolumab, as well as trends in postoperative outcomes according to NAC use, are limited.

To address these evidence gaps, we analyzed systemic treatment patterns among patients with MIBC who underwent RC in Japan from 2013 to 2024 using a nationwide large administrative database. We examined three periods defined by major clinical milestones (pre‐RARC, post‐RARC, and the ICI era) to characterize real‐world adoption of NAC and AT, chemotherapy regimen selection, and postoperative mortality.

## 2 Methods

### 2.1 Study Design

**FIGURE 1:**
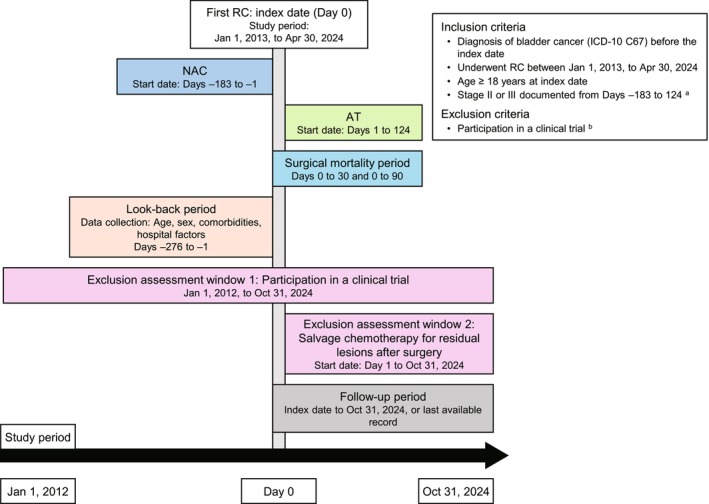
Study design. Days were counted relative to the index date (Day 0). NAC was assessed during the prespecified NAC window (Days −183 to −1), and AT was assessed during the prespecified AT window (Days 1 to 124). A look‐back period, surgical mortality period, and follow‐up period were applied. ^a^TNM stage classification was assigned according to the UICC TNM staging system, using the 6th/7th editions [15, 16] or the 8th edition [17]. ^b^Clinical trial participation based on MDV hospital records. AT, adjuvant therapy; ICD‐10, International Classification of Diseases, 10th Revision; MDV, Medical Data Vision; NAC, neoadjuvant chemotherapy; RC, radical cystectomy; TNM, tumor–node–metastasis staging system; UICC, Union for International Cancer Control.

This retrospective observational cohort study used the Medical Data Vision (MDV) database, which includes health claims and administrative data or Diagnosis Procedure Combination (DPC) data, from approximately 500 medical institutions in Japan (as of April 2022) [13, 14]. The extraction period was from January 1, 2012, to October 31, 2024 (Figure 1). The date of RC was defined as the index date (Day 0). Patients were followed until the last available record or until October 31, 2024.

### 2.2 Study Population

Patients were eligible if they had a diagnosis of bladder cancer (International Classification of Diseases, 10th Revision [ICD‐10] code C67) before the index date, who underwent RC between January 1, 2013, and April 30, 2024, were aged ≥ 18 years on the index date, and had a record of stage II or III disease (tumor–node–metastasis [TNM] classification). Patients who participated in a clinical trial were excluded. NAC and AT were defined relative to the date of RC (index date, Day 0). NAC was defined as systemic therapy with a start date between Days −183 and −1, and AT was defined as systemic therapy with a start date between Days 1 and 124 (Figure 1).

### 2.3 Data Collection

The MDV database provides ICD‐10 diagnosis codes, Japanese procedure codes, and prescription information coded based on receipt and Anatomical Therapeutic Chemical (ATC) classifications. Demographic characteristics, hospital size, and TNM stage (6th/7th [15, 16] or 8th [17] edition) were extracted. Data were collected on the RC procedure type, NAC and AT regimens received, dates of exposure, treatment sequencing, and deaths from the surgery.

Table S1 summarizes the eligible NAC and AT regimens and the identification rules. All regimens were identified based on the regimen used in the first cycle.

### 2.4 Endpoints

The primary endpoint was the proportion of patients receiving each peri‐RC treatment pattern during the three calendar periods defined by major clinical milestones (pre‐RARC: 2013–2017; post‐RARC: 2018–2021; ICI era: 2022–2024), specifically NAC + RC + AT, NAC + RC, RC + AT, or RC alone. Secondary endpoints included the distribution of NAC regimens and RC alone by period and treatment sequences in 2022–2024. Additional endpoints included 30‐ and 90‐day post‐RC mortality, evaluated for the overall NAC group, individual NAC regimens, and the RC alone group.

### 2.5 Statistical Analysis

All analyses conducted in the full analysis set (FAS) were descriptive. Categorical variables were summarized as counts and percentages, and continuous variables as medians with minimum and maximum values. Mortality rates were calculated for the 30‐ and 90‐day post‐RC windows. Treatment sequences in 2022–2024 were visualized using a Sankey plot. No formal hypothesis testing was performed. Analyses were conducted using SAS Viya, version 3.5 (SAS Institute Inc., Cary, North Carolina, USA) and R, version 4.4.1 (R Foundation for Statistical Computing, Vienna, Austria).

## 3 Results

### 3.1 Patient Characteristics by Study Period

**FIGURE 2:**
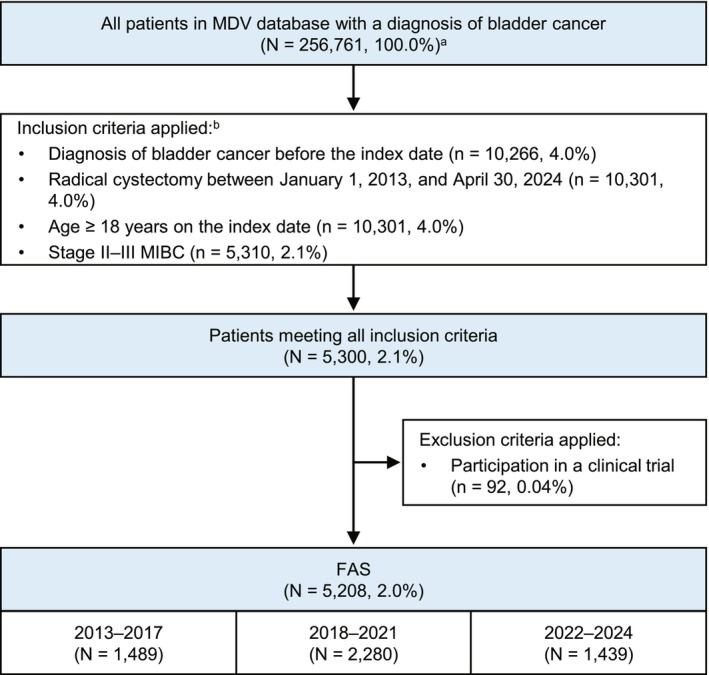
Patient disposition. ^a^Denominator is the number of patients in “All patients.” ^b^Patients may be counted in multiple inclusion criteria. FAS, full analysis set; MDV, Medical Data Vision; MIBC, muscle‐invasive bladder cancer.

Of 256 761 patients with a diagnosis of bladder cancer, 10 301 (4.0%) underwent RC between January 1, 2013, and April 30, 2024; this criterion accounted for the largest decrease in the study population size. After applying all other inclusion and exclusion criteria, 5 208 patients satisfied the study eligibility criteria and were included in the FAS, comprising 1 489 patients in 2013–2017, 2 280 patients in 2018–2021, and 1 439 patients in 2022–2024 (Figure 2).

Across the three study periods, the median age was stable over time, ranging from 71 to 73 years (Table 1). Patients who received any NAC (NAC + RC and NAC + RC + AT) were numerically younger than those who underwent RC alone in all three periods (Table S2). Stage II disease was less common in patients who received any NAC (NAC + RC and NAC + RC + AT) than in those who underwent RC alone, whereas stage III disease was more common in patients treated with NAC + RC + AT than in those treated with RC alone (Table S2).

**TABLE 1:** Baseline patient characteristics by study period.

Characteristic	2013–2017 (*N* = 1 489)	2018–2021 (*N* = 2 280)	2022–2024 (*N* = 1 439)
Age, median (min, max), years	71 (26, 91)	72 (29, 95)	73 (30, 93)
Sex, male	1 145 (76.9)	1 780 (78.1)	1 067 (74.1)
Surgery type			
Open RC	Not recorded	1 286 (56.4)	518 (36.0)
LRC/RARC	Not recorded	994 (43.6)	921 (64.0)
Smoking history	753 (50.6)	1 180 (51.8)	720 (50.0)
TNM stage^a^			
Stage II (6th/7th ed.)	884 (59.4)	424 (18.6)	67 (4.7)
Stage III (6th/7th ed.)	604 (40.6)	262 (11.5)	34 (2.4)
Stage II (8th ed.)	0	918 (40.3)	786 (54.6)
Stage IIIA (8th ed.)	1 (0.1)	596 (26.1)	485 (33.7)
Stage IIIB (8th ed.)	0	80 (3.5)	67 (4.7)
Hospital size			
< 200 beds	28 (1.9)	59 (2.6)	24 (1.7)
200–499 beds	858 (57.6)	1 093 (47.9)	620 (43.1)
≥ 500 beds	603 (40.5)	1 128 (49.5)	795 (55.2)
Time from the start date of the final NAC to surgery, days^b^			
*n*	546	1 306	1 024
Median (min, max)	43.0 (17, 177)	42.0 (12, 174)	46.0 (11, 170)
Time from surgery to the start of AT, days			
*n*	293	338	354
Median (min, max)	58.0 (18, 124)	57.0 (15, 124)	62.0 (21, 124)

*Note:* Data are presented as *n* (%), unless specified otherwise.

Abbreviations: AT, adjuvant therapy; LRC, laparoscopic radical cystectomy; NAC, neoadjuvant chemotherapy; RARC, robot‐assisted radical cystectomy; RC, radical cystectomy; TNM, tumor–node–metastasis staging system; UICC, Union for International Cancer Control.

^a^ TNM stage classification was assigned according to the UICC TNM staging system, using the 6th/7th editions [15, 16] or the 8th edition [17].

^b^ The time to surgery from the start date of final NAC was calculated for patients who received the following NAC regimens: standard‐, reduced‐, or split‐dose gemcitabine + cisplatin; gemcitabine‐carboplatin; or methotrexate + vinblastine + doxorubicin + cisplatin (conventional or dose‐dense). This excludes patients who received other regimens and patients without NAC.

### 3.2 Treatment Patterns

Surgical practice evolved markedly across the three study periods: laparoscopic radical cystectomy (LRC) or RARC increased from 43.6% in 2018–2021 to 64.0% in 2022–2024. Data for 2013–2017 were not evaluated.

**FIGURE 3:**
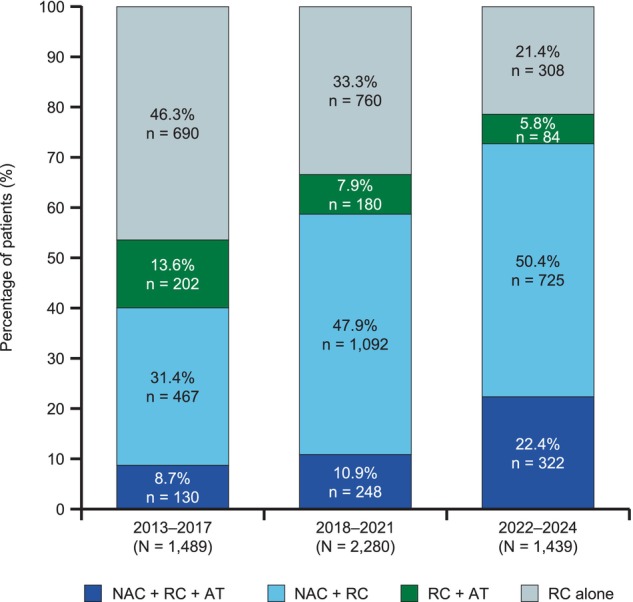
Perioperative treatment patterns over time (2013–2024). AT, adjuvant therapy; NAC, neoadjuvant chemotherapy; RC, radical cystectomy.

Perioperative systemic therapy increased substantially over time (Figure 3). NAC + RC increased from 31.4% of patients in 2013–2017 to 47.9% in 2018–2021 and 50.4% in 2022–2024. NAC + RC + AT also increased, from 8.7% in 2013–2017 and 10.9% in 2018–2021 to 22.4% in 2022–2024. Conversely, RC alone decreased from 46.3% in 2013–2017 to 33.3% in 2018–2021 and to 21.4% in 2022–2024.

Standard‐dose GC and reduced‐dose GC were consistently the most frequently used regimens across all periods, accounting for 34.9% and 34.6% of patients who received NAC in 2022–2024 (Table 2). The use of dose‐modified GC or carboplatin‐based regimens (reduced‐dose GC, split‐dose GC, and gemcitabine + carboplatin [G‐carboplatin]) exceeded the use of standard‐dose GC in every period; the use of G‐carboplatin increased from 13.6% in 2013–2017 to 16.9% in 2018–2021 and to 23.4% in 2022–2024. The use of split‐dose GC and methotrexate + vinblastine + doxorubicin + cisplatin (MVAC) remained low, each accounting for < 1% of patients in all periods. The use of dd‐MVAC slightly increased to 3.9% in 2022–2024.

**TABLE 2:** Proportions of patients receiving each NAC treatment^a^.

Regimen type	2013–2017 (*N* = 597)	2018–2021 (*N* = 1 340)	2022–2024 (*N* = 1 047)
Standard‐dose GC	220 (36.9)	506 (37.8)	365 (34.9)
Reduced‐dose GC	238 (39.9)	549 (41.0)	362 (34.6)
Split‐dose GC	2 (0.3)	10 (0.7)	7 (0.7)
G‐carboplatin	81 (13.6)	226 (16.9)	245 (23.4)
MVAC	4 (0.7)	3 (0.2)	4 (0.4)
dd‐MVAC	1 (0.2)	12 (0.9)	41 (3.9)
Other regimens	51 (8.5)	34 (2.5)	23 (2.2)

*Note:* Data are presented as *n* (%).

Abbreviations: dd‐MVAC, dose‐dense methotrexate + vinblastine + doxorubicin + cisplatin; G‐carboplatin, gemcitabine + carboplatin; GC, gemcitabine + cisplatin; MVAC, methotrexate + vinblastine + doxorubicin + cisplatin; NAC, neoadjuvant chemotherapy.

^a^ Among patients who received NAC + radical cystectomy ± adjuvant therapy.

**FIGURE 4:**
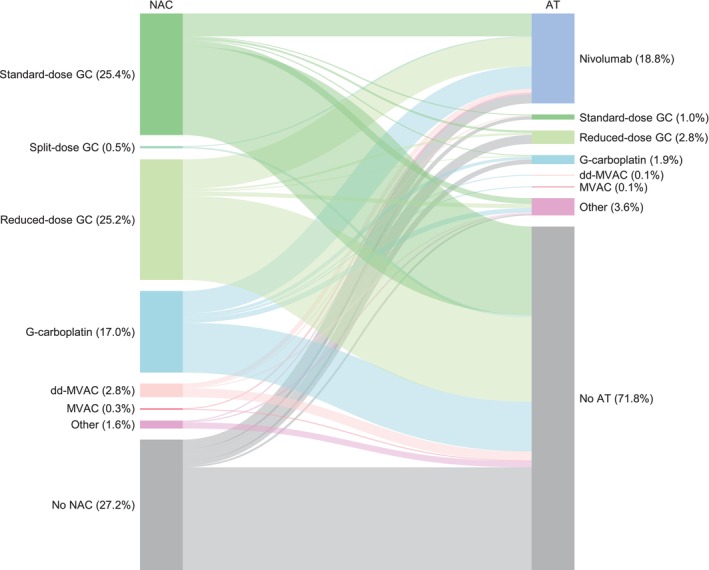
Flow of perioperative systemic therapy (2022–2024). AT, adjuvant therapy; dd‐MVAC, dose‐dense methotrexate + vinblastine + doxorubicin + cisplatin; G‐carboplatin, gemcitabine + carboplatin; GC, gemcitabine + cisplatin; MVAC, methotrexate + vinblastine + doxorubicin + cisplatin; NAC, neoadjuvant chemotherapy.

Figure 4 shows treatment flow patterns for 2022–2024. Among the 1 439 patients who underwent RC in this study period, 28.2% (*n* = 406) received any AT and 18.8% (*n* = 270) received adjuvant nivolumab. Among patients who received adjuvant nivolumab, 242 patients had received NAC and 28 patients had not received NAC.

### 3.3 Postoperative Mortality

Across the three study periods, 30‐ and 90‐day mortality rates after RC remained low in both the NAC and no‐NAC groups (Table 3). In the no‐NAC group, the 30‐day mortality rates were 0.3% in 2013–2017, 0.6% in 2018–2021, and 1.5% in 2022–2024. Among patients who received any NAC, the 30‐day mortality rates were 0.0% in 2013–2017, 0.1% in 2018–2021, and 0.2% in 2022–2024. The 90‐day mortality rates in the no‐NAC group were 2.1% in 2013–2017, 2.7% in 2018–2021, and 3.3% in 2022–2024. Among patients who received any NAC, the 90‐day mortality rates were 0.5% in 2013–2017, 1.4% in 2018–2021, and 0.9% in 2022–2024.

**TABLE 3:** 30‐ and 90‐day postoperative mortality by neoadjuvant therapy regimen and period.

Treatment pattern	2013–2017		2018–2021		2022–2024	
	30‐day mortality, *n*/*N* (%)	90‐day mortality, *n*/*N* (%)	30‐day mortality, *n*/*N* (%)	90‐day mortality, *n*/*N* (%)	30‐day mortality, *n*/*N* (%)	90‐day mortality, *n*/*N* (%)
NAC total	0/597	3/597 (0.5)	2/1 340 (0.1)	19/1 340 (1.4)	2/1 047 (0.2)	9/1 047 (0.9)
Standard‐dose GC	0/220	1/220 (0.5)	1/506 (0.2)	8/506 (1.6)	0/365	3/365 (0.8)
Reduced‐dose GC	0/238	1/238 (0.4)	0/549	9/549 (1.6)	1/362 (0.3)	2/362 (0.6)
Split‐dose GC	0/2	0/2	0/10	0/10	0/7	0/7
G‐carboplatin	0/81	0/81	1/226 (0.4)	2/226 (0.9)	1/245 (0.4)	4/245 (1.6)
MVAC	0/4	0/4	0/3	0/3	0/4	0/4
dd‐MVAC	0/1	0/1	0/12	0/12	0/41	0/41
Other regimens	0/51	1/51 (2.0)	0/34	0/34	0/23	0/23
No NAC	3/892 (0.3)	19/892 (2.1)	6/940 (0.6)	25/940 (2.7)	6/392 (1.5)	13/392 (3.3)

Abbreviations: dd‐MVAC, dose‐dense methotrexate + vinblastine + doxorubicin + cisplatin; G‐carboplatin, gemcitabine + carboplatin; GC, gemcitabine + cisplatin; MVAC, methotrexate + vinblastine + doxorubicin + cisplatin; NAC, neoadjuvant chemotherapy.

## 4 Discussion

To our knowledge, this nationwide observational study is the first to characterize temporal changes in perioperative systemic treatment and postoperative mortality in Japanese patients with stage II–III MIBC undergoing RC from 2013 to 2024 using a large administrative database. Multiple new perioperative treatment options for MIBC have been introduced in recent years; however, comprehensive large‐scale real‐world data describing how perioperative systemic therapy has changed over time in Japan and how these changes relate to postoperative mortality have been limited. The use of NAC increased over time, with a corresponding increase in perioperative systemic therapy intensity in the most recent period (including uptake of adjuvant nivolumab). Across all study periods, 30‐ and 90‐day postoperative mortality remained low in both the NAC and no‐NAC groups, indicating that perioperative safety was preserved as systemic therapy evolved.

In Japan, perioperative treatment patterns for MIBC have changed substantially over the past decades. Earlier nationwide analyses reported limited use of perioperative systemic therapy, with NAC and AT rates of 5.5% and 3.3% in 2008 and 2011, respectively [7], increasing to 56% for NAC in 2013–2019 [18]. In our study, NAC + RC and NAC + RC + AT became increasingly common; however, RC alone was still used in some patients, suggesting the possibility of differential use according to patient characteristics such as age and disease stage. NAC use increased during the study periods, reflecting ongoing changes in clinical practice. These shifts likely reflect the revision of guideline recommendations for perioperative chemotherapy in MIBC, improvements in the management of chemotherapy‐related adverse events, and the increasing adoption of RARC with its lower postoperative complication profile, which have facilitated broader real‐world adoption [19]. The increasing use of NAC in Japan contrasts with contemporary real‐world data from Western countries, where uptake has been comparatively lower. For instance, recent European population‐based studies have reported steady NAC utilization rates of approximately 29% to 31% among patients undergoing RC in Finland and the Netherlands [20, 21]. Similarly, a contemporary real‐world cohort in the United States demonstrated a NAC utilization rate of 46% [22]. The robust uptake in our Japanese cohort likely reflects the proactive adoption of revised national guidelines and specific regional clinical practices.

The use of NAC substantially increased across the study period, and included cisplatin‐based regimens recommended in international guidelines [23] as well as less intensive regimens. One of those regimens, dd‐MVAC, was infrequently used in Japan in the study period, which might be partly due to concerns about tolerability and adverse events. Indeed, a prior study of Japanese patients suggested significant adverse events hindered the ability to complete the target number of cycles [24]. Previous studies have defined “suboptimal NAC” as reduced‐dose cisplatin, fewer than three cycles of cisplatin‐based therapy, or non‐cisplatin regimens [25]. These studies have shown that suboptimal NAC is often administered to patients with impaired renal function, higher comorbidity burden, or poorer performance status. Although suboptimal NAC is associated with consistently poorer oncologic outcomes compared with optimal cisplatin‐based therapy, we found that reduced‐dose GC, split‐dose GC, and G‐carboplatin remained common throughout the study period, underscoring ongoing challenges in real‐world MIBC populations. This high prevalence of dose‐modified GC or carboplatin‐based regimens in Japan also highlights distinct regional differences in treatment selection. Real‐world data from the United States show that GC (58%) and MVAC (30%) are the predominant regimens [22]. Similarly, in Finland, GC accounts for 92% of all administered NAC [21]. The comparatively wider use of dose‐modified GC or carboplatin‐based regimens in our study underscores the specific challenges of managing cisplatin‐ineligible MIBC populations in real‐world Japanese practice.

Evidence‐based perioperative treatment options for patients with MIBC are evolving. The phase III NIAGARA trial provided new clinical evidence for durvalumab plus split‐dose GC among patients with borderline renal function [26], a population lacking robust evidence. More recently, the phase III EV‐303/KEYNOTE‐905 trial evaluated perioperative enfortumab vedotin plus pembrolizumab in cisplatin‐ineligible or cisplatin‐refusing patients with MIBC [27]. Taken together, the findings of these trials suggest that evidence‐based perioperative treatment options for patients who are ineligible for standard cisplatin‐based NAC are likely to expand in the near future.

The use of AT also increased between 2018 to 2021 and 2022 to 2024. Adjuvant nivolumab was the most commonly used AT. Of the 270 patients who received adjuvant nivolumab, the majority (*n* = 242) had previously received NAC, which is consistent with the 2022 update of the Japanese guidelines, recommending postoperative nivolumab in patients who previously received cisplatin‐based NAC [28]. These findings suggest that the introduction of adjuvant nivolumab may have contributed to the increased NAC use. The clinical experience gained with nivolumab, particularly in adverse event recognition and management, should inform efficient implementation of forthcoming ICI‐based perioperative treatment approaches.

Postoperative mortality in this study was consistently low. Importantly, mortality was numerically lower in the NAC group than in the no‐NAC group, indicating that the addition of NAC did not compromise perioperative safety. The data for both groups also align with a prior Japanese series, reporting 30‐ and 90‐day mortality after RC of 0.8% and 2.0%, respectively [29]. With the increasing use of immunotherapy, other clinical outcomes such as treatment‐related toxicities, treatment completion rates, and immune‐related adverse events are highly important and need to be evaluated to understand their full impact [30]. However, these data were not available for this study. In this treatment setting, perioperative safety is the most important factor to consider in the treatment strategy. The absence of increased mortality despite greater systemic therapy use likely reflects appropriate treatment selection and improvements in perioperative care. These findings underscore that, as treatment intensity is escalated and new treatments are introduced, rigorous patient selection and optimizing perioperative care will remain essential to preserve safety.

This study has limitations inherent to the retrospective analysis of a claims‐based database. First, the dataset lacks granular clinical information, such as renal function and performance status, and its findings may have limited generalizability to hospitals not included in the MDV database. Moreover, because the number of participating institutions expanded over time, the observed increase in patients undergoing RC may reflect this database growth rather than a true epidemiological increase in procedural volume. Second, the use of multiple TNM classification systems over the observation period may have introduced heterogeneity and potential misclassification. Staging relied entirely on the specific version of the code entered by each institution at the time of treatment, and potential input errors cannot be definitively excluded or adjusted for. Third, treatment and procedural coding lacked sufficient specificity. Surgical codes do not reliably distinguish between laparoscopic and robot‐assisted approaches, nor can they confirm with sufficient specificity if concomitant procedures (e.g., nephroureterectomy, lymph node dissection, or specific urinary diversions) were performed. Similarly, chemotherapy regimens were categorized solely by the initial cycle, which may not adequately reflect complex, clinically appropriate dose modifications made over the subsequent course of therapy. Due to these classification limits and unmeasured confounders, we could not accurately evaluate the impact of specific surgical approaches or longitudinal regimen adjustments on postoperative mortality. Finally, we did not assess the length of hospital stay or complications owing to the challenges of assessing these in the MDV database. Events occurring after inter‐hospital transfers may have been incompletely captured. In addition, short‐term readmissions are often merged into a single admission in the MDV database, which can artificially prolong the calculated length of stay, and monthly diagnostic coding prevents the reliable differentiation of preexisting comorbidities from true postoperative complications. The capture of post‐RC mortality was restricted to in‐hospital deaths at participating facilities, potentially underestimating deaths occurring elsewhere (including unaffiliated facilities or outside the hospital system). However, because treatment for MIBC is typically initiated and continued within the same institution, the risk of underestimating mortality within 30 or 90 days after RC is likely minimal.

In conclusion, systemic treatment patterns for MIBC in Japan changed substantially from 2013 to 2024, with increasing use of NAC and NAC + RC + AT, alongside persistent use of dose‐modified GC or carboplatin‐based regimens among cisplatin‐ineligible patients. Postoperative mortality remained low, despite the higher proportion of patients receiving NAC, supporting continued guideline‐recommended NAC use.

## Author Contributions

**Eiji Kikuchi:** conceptualization, methodology, writing – review and editing. **Jumpei Tokumaru:** conceptualization, data curation, methodology, project administration, visualization, writing – original draft, writing – review and editing. **Daisuke Kawai:** conceptualization, data curation, methodology, project administration, writing – review and editing. **Ryotaro Ide:** data curation, formal analysis, investigation, methodology, validation, writing – review and editing. **Hiroshi Komazaki:** data curation, formal analysis, investigation, methodology, validation, writing – review and editing. **Hiroshi Kitagawa:** methodology, supervision, writing – review and editing. **Junichi Inokuchi:** methodology, conceptualization, writing – review and editing.

## Funding

This work was supported by AstraZeneca K.K.

## Ethics Statement

This study was conducted in accordance with the Declaration of Helsinki, the Ethical Guidelines for Medical and Biological Research Involving Human Subjects, Good Pharmacoepidemiology Practices, and all applicable legislation governing non‐interventional observational research. The study protocol was reviewed and approved by the MINS Institutional Review Board, an independent, nonprofit ethics committee (MINS‐REC‐250202; approved January 9, 2025).

## Consent

The analysis used de‐identified data extracted from a commercially available administrative claims database; therefore, informed consent was not required under the Ethical Guidelines.

## Conflicts of Interest

Eiji Kikuchi reports grants or contracts (to the institution) from Astellas, AstraZeneca, Bristol Myers Squibb, Chugai, Janssen, Merck Biopharma, MSD, Nippon Kayaku, and Taiho; consulting fees from Astellas, AstraZeneca, Bristol Myers Squibb, Chugai, Daiichi Sankyo, Ferring, Janssen, Merck Biopharma, MSD, Nippon Kayaku, and Pfizer; and payment or honoraria (personal) for lectures, presentations, speakers bureaus, manuscript writing, or educational events from Astellas, AstraZeneca, Bristol Myers Squibb, Chugai, Ferring, Janssen, Merck Biopharma, MSD, and Nippon Kayaku. Junichi Inokuchi reports grants or contracts from Merck & Co. and honoraria for speakers bureaus from Astellas Pharma, Ono Pharmaceutical, Merck & Co, Takeda Pharmaceutical, and AstraZeneca. Jumpei Tokumaru, Daisuke Kawai, Ryotaro Ide, Hiroshi Komazaki, and Hiroshi Kitagawa are employees of AstraZeneca K.K.

## Supporting information


**Table S1:** Definitions of perioperative systemic treatment regimens used for NAC and AT in the MDV database.
**Table S2:** Baseline patient characteristics by study period.

## Data Availability

The data used in this study cannot be shared with external researchers due to the terms of the research contract with Medical Data Vision. Researchers may contact Medical Data Vision directly for all data requests (https://en.mdv.co.jp/).
