# Supplementary material for: Perioperative Systemic Treatment Patterns and Postoperative Mortality Among Patients With Muscle‐Invasive Bladder Cancer Undergoing Radical Cystectomy in Japan: WAKKA‐MIBC Study
Source: Int J Urol. 2026 Jul 25;33(7):e70565. doi: 10.1111/iju.70565 (PMC13401184; doi:10.1111/iju.70565)
Supplement: Supplementary file 1 — Table S1: Definitions of perioperative systemic treatment regimens used for NAC and AT in the MDV database. Table S2: Baseline patient characteristics by study period. [file IJU-33-0-s001.docx]

## Supporting information

## Table S1.

Definitions of perioperative systemic treatment regimens used for NAC and AT in the MDV database.

| **Treatment setting** | **Regimen name** | **Abbreviation / core drugs** | **Key identification rules in MDV claims data^a^** |
| --- | --- | --- | --- |
| NAC or AT | Standard-dose gemcitabine–cisplatin | Standard-dose GC (gemcitabine + cisplatin) | Gemcitabine and cisplatin prescribed in the NAC window (Days −183 to −1) or AT window (Days 1–124). Day 1 defined as the first gemcitabine prescription in the cycle. Gemcitabine given on Day 1 and Day 8 (± Day 15); cisplatin given on Day 1 or 2 of cycle 1. Cisplatin dose at cycle-1 Day 1/2 ≥ 95% of the standard dose (70 mg/m^2^ × BSA, calculated from height/weight). Within each 3-week period, cisplatin not prescribed twice (i.e., not split-dose). Cycles counted based on repeated cisplatin prescriptions at ≥ 21-day intervals. |
|  | Split-dose gemcitabine–cisplatin | Split-dose GC (gemcitabine + cisplatin) | Gemcitabine and cisplatin prescribed together on 2 occasions within a 3-week period in the NAC or AT window. Day 1 defined as the first day on which both drugs are prescribed; second administration between Day 1+1 and Day 1+20. Within each 3-week period, cisplatin prescribed twice (split dose). Classified as split-dose GC regardless of later dose reductions/omissions. Cycles counted based on repeated cisplatin prescriptions at ≥ 21-day intervals. |
|  | Reduced-dose gemcitabine–cisplatin | Reduced-dose GC (gemcitabine + cisplatin) | Same scheduling as standard-dose GC (gemcitabine on Day 1 and Day 8 (± Day 15); cisplatin on Day 1 or 2), but cisplatin dose at cycle-1 Day 1/2 < 95% of the standard dose (70 mg/m^2^ × BSA). Within each 3-week period, cisplatin not prescribed twice. Classified as reduced-dose GC even if subsequent cycles are modified. Cycles counted based on repeated cisplatin prescriptions at ≥ 21-day intervals. |
|  | Unknown-dose gemcitabine–cisplatin | Unknown-dose GC (gemcitabine + cisplatin) | Gemcitabine and cisplatin co-prescribed in a pattern compatible with GC in the NAC or AT window, but BSA cannot be calculated and/or dose or scheduling does not meet the definitions of standard-dose, split-dose, or reduced-dose GC. Day 1 identified using the same rules as for GC regimens. Cycles counted according to the GC definitions. |
|  | Gemcitabine–carboplatin | G-carboplatin (gemcitabine + carboplatin) | Gemcitabine and carboplatin prescribed in the NAC or AT window. Day 1 defined as the first gemcitabine prescription in the cycle. Gemcitabine given on Day 1 and Day 8 (± Day 15); carboplatin given on Day 1 or 2 of cycle 1. Day 1 in NAC and AT identified using the same “Day X / Day X+1” rule as GC. Presence of G-carboplatin defined by gemcitabine + carboplatin prescriptions on Day 1 and/or Day 2 of cycle 1. Cycles counted based on repeated carboplatin prescriptions at ≥ 21-day intervals. |
|  | Dose-dense MVAC | dd-MVAC (methotrexate + vinblastine + doxorubicin + cisplatin + pegfilgrastim ± gemcitabine) | Methotrexate, vinblastine, doxorubicin, cisplatin and pegfilgrastim (with or without gemcitabine) prescribed in the NAC or AT window. Day 1 defined as the methotrexate prescription day. Vinblastine (and/or doxorubicin) and cisplatin prescribed on Day 1+1; pegfilgrastim on any of Days 1+1 to 1+4. Presence of dd-MVAC determined from this pattern in Days 1–5 of cycle 1. Cycles counted based on repeated cisplatin prescriptions at ≥ 14-day intervals. |
|  | Conventional MVAC | MVAC (methotrexate + vinblastine + doxorubicin + cisplatin) | Methotrexate, vinblastine, doxorubicin and cisplatin prescribed in the NAC or AT window. Day 1 defined as the first methotrexate prescription in the cycle. Typical pattern: methotrexate on Days 1, 15, and 22; vinblastine on Days 2, 15, and 22; doxorubicin and cisplatin on Day 2. Presence of MVAC defined by methotrexate + vinblastine (± doxorubicin) + cisplatin on Day 1 and Day 2 of cycle 1. Cycles counted based on repeated cisplatin prescriptions at ≥ 28-day intervals. |
|  | Other systemic regimens | Other | Any systemic regimen that does not meet the definitions above, including (i) monotherapies with pembrolizumab, avelumab, enfortumab vedotin, docetaxel, paclitaxel, gemcitabine or cisplatin; (ii) combination regimens such as CMV (cisplatin + methotrexate + vinblastine), M-CAVI (methotrexate + carboplatin + vinblastine), GP (gemcitabine + paclitaxel), CP (carboplatin + paclitaxel), M-VECa (methotrexate + vinblastine + epirubicin + carboplatin), PIN (paclitaxel + ifosfamide + nedaplatin), PN (paclitaxel + nedaplatin); and (iii) cisplatin + radiotherapy, defined as cisplatin prescribed within 28 days after the start of radiotherapy. Identification is based on Anatomical Therapeutic Chemical (ATC) and receipt codes specified in the study protocol appendices. |
| AT only | Nivolumab monotherapy | Nivolumab | Nivolumab prescribed as monotherapy in the AT window (Days 1–124). Day 1 defined as the first nivolumab prescription date closest to Day 1. Subsequent cycles not counted for this analysis. |

^a^General rules applied to all regimens: NAC was defined as systemic treatment initiated between Day −183 and Day −1 relative to the date of RC (index date). AT was defined as systemic treatment initiated between Day 1 and Day 124 after RC. “Day 1” in this table refers to the start date of each treatment cycle (not the index date). For GC-based, G-carboplatin, dd-MVAC and MVAC regimens, the number of cycles was counted based on repeated cisplatin or carboplatin prescriptions separated by the minimum interval defined for each regimen; AT was considered complete at death or if there was a gap of ≥ 6 months (183 days) between cycles.

AT, adjuvant therapy; ATC, Anatomical Therapeutic Chemical; BSA, body surface area; CMV, cisplatin + methotrexate + vinblastine; CP, carboplatin + paclitaxel; dd-MVAC, dose-dense methotrexate + vinblastine + doxorubicin + cisplatin; G-carboplatin, gemcitabine + carboplatin; GC, gemcitabine + cisplatin; GP, gemcitabine + paclitaxel; MDV, Medical Data Vision; M-CAVI, methotrexate + carboplatin + vinblastine; M-VECa, methotrexate + vinblastine + epirubicin + carboplatin; MVAC, methotrexate + vinblastine + doxorubicin + cisplatin; NAC, neoadjuvant chemotherapy; PIN, paclitaxel + ifosfamide + nedaplatin; PN, paclitaxel + nedaplatin; RC, radical cystectomy.

## Table S2.

Baseline patient characteristics by study period.

| **Characteristic** | **2013–2017 (N = 1 489)** | | | | **2018–2021 (N = 2 280)** | | | | **2022–2024 (N = 1 439)** | | | |
| --- | --- | --- | --- | --- | --- | --- | --- | --- | --- | --- | --- | --- |
|  | **NAC + RC + AT (n = 130)** | **NAC** **+ RC (n = 467)** | **RC + AT (n = 202)** | **RC alone (n = 690)** | **NAC + RC + AT (n = 248)** | **NAC + RC (n = 1,092)** | **RC + AT (n = 180)** | **RC alone (n = 760)** | **NAC + RC + AT (n = 322)** | **NAC + RC (n = 725)** | **RC + AT (n = 84)** | **RC alone (n = 308)** |
| Age, years |  |  |  |  |  |  |  |  |  |  |  |  |
| Median | 69.0 | 70.0 | 70.0 | 73.0 | 70.0 | 71.0 | 72.0 | 76.0 | 71.0 | 73.0 | 73.5 | 76.0 |
| IQR | (64, 74) | (65, 75) | (65, 75) | (67, 78) | (65, 75) | (66, 76) | (66, 76) | (70, 81) | (65, 75) | (68, 77) | (68, 79) | (72, 82) |
| Min, max | (46, 82) | (30, 88) | (26, 82) | (39, 91) | (39, 86) | (29, 90) | (45, 85) | (39, 95) | (42, 85) | (30, 87) | (47, 87) | (40, 93) |
| Male | 103 (79.2) | 352 (75.4) | 161 (79.7) | 529 (76.7) | 199 (80.2) | 872 (79.9) | 143 (79.4) | 566 (74.5) | 247 (76.7) | 550 (75.9) | 61 (72.6) | 209 (67.9) |
| Surgery type |  |  |  |  |  |  |  |  |  |  |  |  |
| Open RC | Not recorded | Not recorded | Not recorded | Not recorded | 145 (58.5) | 559 (51.2) | 121 (67.2) | 461 (60.7) | 97 (30.1) | 253 (34.9) | 39 (46.4) | 129 (41.9) |
| LRC/RARC | Not recorded | Not recorded | Not recorded | Not recorded | 103 (41.5) | 533 (48.8) | 59 (32.8) | 299 (39.3) | 225 (69.9) | 472 (65.1) | 45 (53.6) | 179 (58.1) |
| Smoking history | 69 (53.1) | 240 (51.4) | 103 (51.0) | 341 (49.4) | 143 (57.7) | 595 (54.5) | 88 (48.9) | 354 (46.6) | 171 (53.1) | 368 (50.8) | 37 (44.0) | 144 (46.8) |
| TNM stage |  |  |  |  |  |  |  |  |  |  |  |  |
| Stage II (6th/7th ed.) | 63 (48.5) | 267 (57.2) | 94 (46.5) | 460 (66.7) | 39 (15.7) | 196 (17.9) | 30 (16.7) | 159 (20.9) | 13 (4.0) | 32 (4.4) | 4 (4.8) | 18 (5.8) |
| Stage III (6th/7th ed.) | 66 (50.8) | 200 (42.8) | 108 (53.5) | 230 (33.3) | 38 (15.3) | 114 (10.4) | 21 (11.7) | 89 (11.7) | 4 (1.2) | 16 (2.2) | 1 (1.2) | 13 (4.2) |
| Stage II (8th ed.) | 0 | 0 | 0 | 0 | 59 (23.8) | 469 (42.9) | 41 (22.8) | 349 (45.9) | 119 (37.0) | 449 (61.9) | 34 (40.5) | 184 (59.7) |
| Stage IIIA (8th ed.) | 1 (0.8) | 0 | 0 | 0 | 92 (37.1) | 292 (26.7) | 63 (35.0) | 149 (19.6) | 154 (47.8) | 209 (28.8) | 36 (42.9) | 86 (27.9) |
| Stage IIIB (8th ed.) | 0 | 0 | 0 | 0 | 20 (8.1) | 21 (1.9) | 25 (13.9) | 14 (1.8) | 32 (9.9) | 19 (2.6) | 9 (10.7) | 7 (2.3) |
| Hospital size |  |  |  |  |  |  |  |  |  |  |  |  |
| < 200 beds | 1 (0.8) | 7 (1.5) | 4 (2.0) | 16 (2.3) | 9 (3.6) | 19 (1.7) | 1 (0.6) | 30 (3.9) | 7 (2.2) | 10 (1.4) | 1 (1.2) | 6 (1.9) |
| 200–499 beds | 75 (57.7) | 241 (51.6) | 125 (61.9) | 417 (60.4) | 108 (43.5) | 495 (45.3) | 96 (53.3) | 394 (51.8) | 125 (38.8) | 319 (44.0) | 39 (46.4) | 137 (44.5) |
| ≥ 500 beds | 54 (41.5) | 219 (46.9) | 73 (36.1) | 257 (37.2) | 131 (52.8) | 578 (52.9) | 83 (46.1) | 336 (44.2) | 190 (59.0) | 396 (54.6) | 44 (52.4) | 165 (53.6) |
| Time to surgery from start date of final NAC, days |  |  |  |  |  |  |  |  |  |  |  |  |
| n | 120 | 426 | ‒ | ‒ | 241 | 1,065 | ‒ | ‒ | 317 | 707 | ‒ | ‒ |
| Median | 41.0 | 43.0 | – | – | 44.0 | 42.0 | – | – | 46.0 | 47.0 | – | – |
| Min, max | (18, 141) | (17, 177) | (–) | (–) | (13, 146) | (12, 174) | (–) | (–) | (13, 156) | (11, 170) | (–) | (–) |
| Time to start of AT from surgery, days |  |  |  |  |  |  |  |  |  |  |  |  |
| n | 113 | ‒ | 180 | ‒ | 173 | ‒ | 165 | ‒ | 275 | ‒ | 79 | ‒ |
| Median | 62.0 | – | 55.5 | – | 57.0 | – | 58.0 | – | 62.0 | – | 60.0 | – |
| Min, max | (18, 124) | (–) | (20, 124) | (–) | (15, 124) | (–) | (20, 124) | (–) | (25, 124) | (–) | (21, 120) | (–) |

Data are presented as n (%), unless specified otherwise.

TNM stage classification was assigned according to the UICC TNM staging system, using the 6th/7th editions [1, 2] or the 8th edition [3].

AT, adjuvant therapy; LRC, laparoscopic radical cystectomy; NAC, neoadjuvant chemotherapy; RARC, robot-assisted radical cystectomy; RC, radical cystectomy; TNM, tumor–node–metastasis staging system; UICC, Union for International Cancer Control.

**References**

[1] L. H. Sobin, C. Wittekind, and Union for International Cancer Control. *TNM Classification of Malignant Tumours*, 6th ed. (John Wiley & Sons, Ltd, 2002).

[2] L. H. Sobin, M. K. Gospodarowicz, and C. Wittekind. *TNM Classification of Malignant Tumours*, 7th ed. (John Wiley & Sons Ltd, 2009).

[3] J. D. Brierley, M. K. Gospodarowicz, and C. Wittekind. *TNM Classification of Malignant Tumours*, 8th ed. (John Wiley & Sons, Ltd, 2017).
